# The stunt of stunted silk: A novel pollination control mechanism in maize

**DOI:** 10.1093/plphys/kiaf625

**Published:** 2026-01-28

**Authors:** Siddique I Aboobuckerصديق أبوبكر, Sidramappa C Talekar, Ursula K Frei, Bing Yang, Thomas Lübberstedt

**Affiliations:** Department of Agronomy, Iowa State University, Ames, IA, United States; Department of Agronomy, Iowa State University, Ames, IA, United States; All India Coordinated Maize Improvement Project, Main Agricultural Research Station, University of Agricultural Sciences, Dharwad, Karnataka, India; Department of Agronomy, Iowa State University, Ames, IA, United States; Division of Plant Science and Technology, Bond Life Sciences Center, University of Missouri, Columbia, MO, United States; Donald Danforth Plant Science Center, St. Louis, MO, United States; Department of Agronomy, Iowa State University, Ames, IA, United States

## Abstract

A single-gene mutation inhibits maize silk growth, delivering a novel genetic approach to pollination control with an attractive potential for application in baby corn breeding.

Dear Editor,

Existing pollination control strategies in plants can be widely classified into biological and nonbiological measures. The two main biological control measures are male sterility and gametophytic incompatibility systems (Kempe and Gils 2011). What if there is a genetic pollination control system with an application in “baby corn” breeding?

“Baby corn”—unfertilized young ears—is a specialty corn, consumed as a delicacy. It is grown all over the world with Thailand being the largest producer at an estimated value of ca. $64 million (Singh 2019). The quality of baby corn is the most important driving factor in determining taste and thus it influences baby corn breeding and production. Baby corn quality is reduced by pollination, since pollination leads to large, pithy and woody ears in just 4 to 5 days, compared with unpollinated ears. Therefore, farmers employ manual detasseling, a labor- and cost- intensive process in baby corn cultivation (Miles et al. 2018). Cytoplasmic male sterility system has been explored for baby corn breeding (Pal et al. 2020) but was not effective in all maize germplasm due to the presence of fertility restorer genes in male parents (Gabay-Laughnan et al. 2004) or environmental factors (Weider et al. 2009) or disease susceptibility (Levings 1990). While characterizing genes and their respective mutants for other reasons, we observed a stunted silk phenotype, which could pave the way for an alternative pollination control mechanism to produce baby corn while preventing unwanted pollination. The underlying gene was named *ZmBMF2* (*BUB1/MAD3 Family 2*; Komaki and Schnittger 2017), due to its strikingly similar genomic structure to *AtBMF2* (Fig. 1a) and are 66% similar to each other at the amino acid level. AtBMF2 is a member of the spindle assembly checkpoint (SAC) complex (Komaki and Schnittger 2017) and *BMF2* is expressed throughout plant growth and development in both *Arabidopsis* and maize (Supplementary Figure S1).

**Figure 1.**
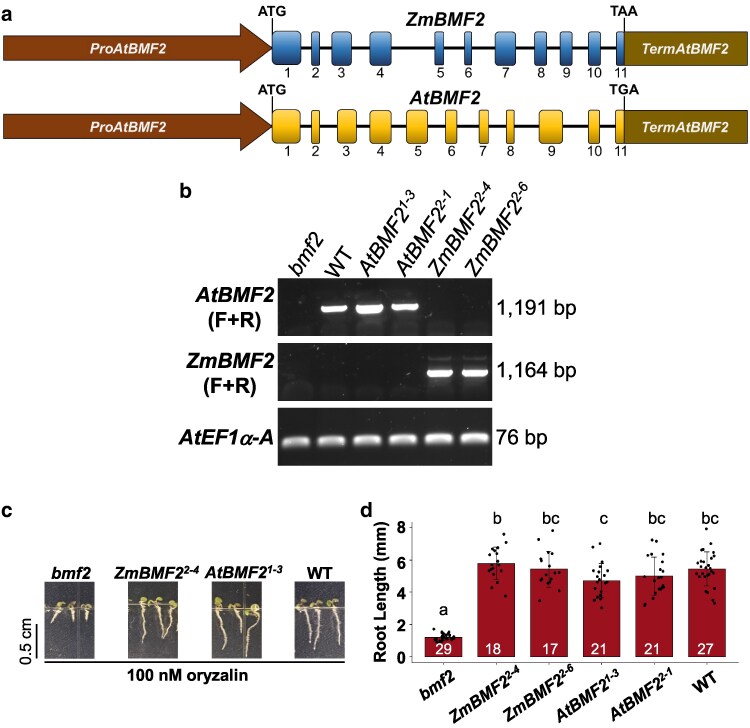
*ZmBMF2* is functionally similar to *AtBMF2*. (a) Cartoon representation of the genomic structure of *ZmBMF2* and *AtBMF2* from the start to stop codons. The promoter and terminator of *AtBMF2* flanking the genomic sequences were used to complement the *Arabidopsis bmf2* mutant. Exons and introns are denoted by boxes and lines, respectively and the exon numbers are also shown. (b) End-point RT-PCR analysis of full length *BMF2* transcript in complemented *Arabidopsis* lines. (c) Representative photographs of 5-day-old *Arabidopsis bmf2* mutant, mutant complemented with *ZmBMF2* or *AtBMF2* constructs (a) and WT (Col-0) grown in 100 nM oryzalin. (d) Root length of *Arabidopsis bmf2* mutant, two independent events (denoted in superscripts) of *bmf2* mutant complemented with *ZmBMF2* or *AtBMF2* and WT (Col-0) grown in 100 nM oryzalin. Data are means ± SD from 17 to 29 seedlings per genotype and the exact number of data points are shown. One-way ANOVA test was conducted followed by Tukey test at a 95% confidence interval. Different letters show statistical significance.

SAC functions during the cell cycle by preventing anaphase progression until all chromosomes are attached to the mitotic spindle by inducing mitotic arrest. Anaphase promoting complex/cyclosome (APC/C), an E3 ubiquitin ligase, is the target for inhibition (Genschik et al., 2013). A mitotic checkpoint complex, made of CDC20 (cell division cycle protein 20, co-activator of the APC/C) and three SAC components: MAD2 (Mitotic Arrest Deficient 2), BUB3 (Budding Uninhibited by Benzimidazole 3), and BUBR1 (BUB-Related 1) are the factors involved in inhibiting APC/C to arrest mitosis. There is a conservation of SAC components between animals and yeast, while plants have a unique SAC molecular architecture. In plants, these are MPS1, BMF1, BMF2, BMF3, MAD1, MAD2, BUB3;1, BUB3;2, and BUB3;3 (Komaki and Schnittger 2017). Further, SAC components in plants do not perform their canonical function as in animals or yeast of inducing a strong delay in mitosis. This raises the question whether the members of SAC in plants have roles other than mitotic arrest.

A *ZmBMF2* genomic fragment (Fig. 1a) was transformed into an *Arabidopsis bmf2* mutant (Supplementary Methods) to determine if *ZmBMF2* can complement the oryzalin sensitive root phenotype (Komaki and Schnittger 2017). Several independent transgenic events were obtained for both *ZmBMF2* and *AtBMF2* genomic constructs. Two events per construct (*ZmBMF2^2-4^, ZmBMF2^2-6^, AtBMF2^1-3^, AtBMF2^2-1^*) with single insertion locus (Supplementary Table S1) were chosen. Full length transcripts of *AtBMF2* and *ZmBMF2* were detected in these lines as expected (Fig. 1b; Supplementary Table S2). There were no apparent morphological differences observed in these transgenic lines. Oryzalin assays showed that *ZmBMF2* construct rescued the *bmf2* root phenotype similar to an *AtBMF2* construct and WT (Fig. 1c), with a mean root length ranging from 4.7 to 5.7 mm in these genotypes compared with 1.2 mm in the *bmf2* mutant (Fig. 1d). Together, these results demonstrate that *ZmBMF2* can complement *Arabidopsis bmf2* mutant root phenotype.

To functionally characterize *ZmBMF2* in maize, we used CRISPR/Cas9 genome editing (Supplementary Methods) in B104 (stiff-stalk heterotic group; Supplementary Figure S2) and obtained four allelic *Zmbmf2* mutants; *Zmbmf2-1, Zmbmf2-2, Zmbmf2-3* and *Zmbmf2-4* (Fig. 2a; Supplementary Figure S3). *Zmbmf2-1,* and *Zmbmf2-4* had nucleotide deletions, *Zmbmf2-2* had an insertion, while *Zmbmf2-3* had an insertion and deletion (Fig. 2a). All these mutations, induced by the first gRNA (gRNA1) due to ineffectiveness of gRNA2, are expected to disrupt the function of the *ZmBMF2* gene, except for *Zmbmf2-4* with an 18-bp in-frame deletion (Fig. 2a). Remarkably, silk length was observed as a qualitative trait, ie, stunted in the mutants (*Zmbmf2-1, Zmbmf2-2* and *Zmbmf2-3)* compared with WT and *Zmbmf2-4,* which is an in-frame deletion, in all summer seasons of 2020 to 2023. In our observations of these mutants in 4 years (2020 to 2023), the silk was not emerging at all outside the husks in all years except in 2023. Photographs from 2023 in Fig. 2 represent the extreme, ie, this was the most silks emerging from husks in these mutants even at the time of harvest (Fig. 2b; Supplementary Figure S4). The CRISPR reagents were introduced - by crossing the transformed B104 carrying Cas9 and gRNAs - into A427, another inbred line in the non-stiff stalk heterotic group, to induce a 1-bp deletion allele *Zmbmf2-5* and the silk length was again reduced in *Zmbmf2-5* (Supplementary Figure S5). Further, to complement the *Zmbmf2-3* mutant, a modified *ZmBMF2* allele (immune from CRISPR targeting; Supplementary Figure S4) was used and a transgenic line carrying the modified *ZmBMF2* transgene as a single insertion was obtained (Supplementary Table S3). The silk length of the complemented line *Zmbmf2-3/ZmBMF2* was restored to WT levels. Silk length was quantified at both the base (“long silk”) and tip (“short silk”) ends of the ears relative to the ear length (Supplementary Figure S4). The estimated mean short silk length was 42% to 45% relative to ear length in *Zmbmf2-1, Zmbmf2-2* and *Zmbmf2-3* mutants, while it ranged from 57% to 60% in the *Zmbmf2-4* mutant, the *Zmbmf2-3/ZmBMF2* genotype and WT (Fig. 2c). Similarly, the long silk length relative to ear length in *Zmbmf2-1, Zmbmf2-2* and *Zmbmf2-3* mutants was 134% to 135%, but it was 148% to 153% in *Zmbmf2-4* mutant, *Zmbmf2-3/ZmBMF2* and WT (Supplementary Figure S4). The stunted silks almost completely abolished seed set in open-pollinated mutant ears. Even the miniscule emergence of silks in the mutants seen in summer 2023 was not sufficient for seed set (Fig. 2b, d). The estimated mean number of kernels per ear in *Zmbmf2-1, Zmbmf2-2* and *Zmbmf2-3* were 6.1, 1.9 and 0.7, respectively (Fig. 2e). In contrast, *Zmbmf2-3/ZmBMF2, Zmbmf2-4* and WT had 268.2, 284.8 and 336.1 kernels per ear on average (Fig. 2e), respectively. The seed set, however, was not impacted in any of the genotypes when the husks were manually removed and ears pollinated (Fig. 2f). The number of kernel rows was also unaffected in the mutants (Supplementary Figure S6). Similarly, the ear length did not show a difference among genotypes with an estimated mean ear length ranging from 13.5 to 14.8 cm (Fig. 2g) ruling out developmental defects in the mutant lines. Together, these results demonstrate that mutations in *ZmBMF2* stunt silk growth without impacting fertility and that the reduction in silk length severely limits seed set.

**Figure 2.**
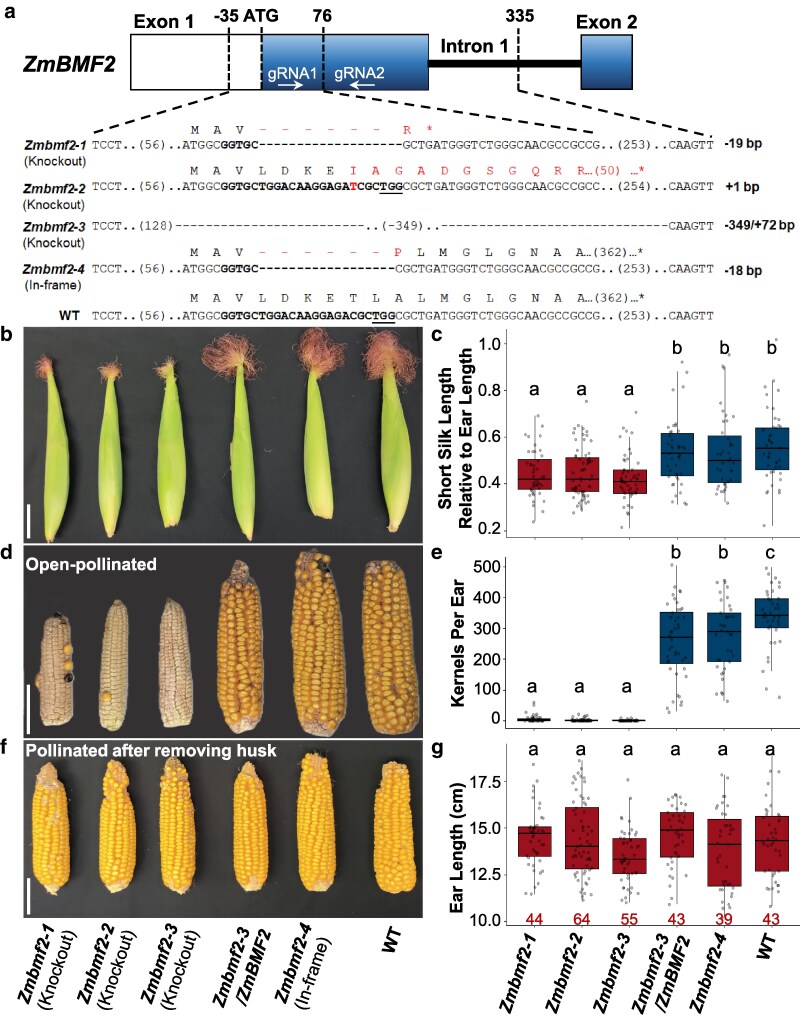
Mutations in *ZmBMF2* reduce silk length without impacting fertility. (a) A cartoon of the *ZmBMF2* gene fragment, the target site for editing and the sequences of the various mutants are shown. gRNA is in bold face and the PAM sequence underlined. The predicted amino acid sequences are also provided. Altered DNA and amino acid sequences are shown. * Stop codon. (b) Representative photographs of ears during the silking stage. (c) Quantification of short silk length relative to ear length from the mutants, complemented line and WT. (d) Representative photographs of ears at the end of the growing season resulting from open-pollination. Images were digitally extracted for comparison. (e) Counts of kernels per ear in the various genotypes. (f) Representative photographs of ears resulting from opening the husk and manually pollinating. (g) Ear length of the different genotypes in cm. Data shown in C, E, G are from two independent experiments conducted in Summer 2022 and 2023. The exact number of data points for each genotype used in C, E, and G are shown below panel G. One-way ANOVA test was conducted followed by Tukey test at a 95% confidence interval. Box plots show the distribution of data points with the median as a center line. The upper (75th percentile) and lower (25th percentile) quartiles are shown by the bounds of boxes, and the whiskers represent the highest and lowest observations. Different letters show statistical significance. Scale bars in B, D, F are 5 cm.

Our discovery of *Zmbmf2* mutants with short silk length holds significant promise as this can overcome the bottlenecks of detasseling in baby corn breeding. The stunted silk limits fertilization (Fig. 2d, e; Supplementary Figure S4) and thereby helps to produce quality ears. Short silk mutants, however, are easy to maintain by opening the husk and manual pollination (Fig. 2f), unlike the *silkless* mutant (Zhao et al. 2018). Further, these mutations can be introduced into desired germplasm by HI-Edit in one step (Kelliher et al. 2019). Future work will be geared toward evaluating taste profile and other agronomic properties in baby corn germplasm with the mutation introgressed and finding pathways for commercialization.

Silk biology is an important area of study due to its role in agricultural productivity. It is poorly studied, however, due to the dearth of mutants. The two genes reported in the first (initiation) and the last (senescence) stages of silk growth dynamics are: *SK1* (Zhao et al. 2018) and *KIL1* (Šimášková et al. 2022). Here, we report a novel mutant in silk growth, the intermediate phase of silk growth dynamics. The identification of *ZmBMF2* in silk growth holds promise in baby corn breeding and opens an avenue to explore the applicability of this mutation in breeding germplasm and to identify the underlying molecular mechanism(s).

## Supplementary Material

kiaf625_Supplementary_Data

## Data Availability

All data are available in the main text or the supplementary materials.

## Supplementary material

The following materials are available in the online version of this article.

**Supplementary Figure S1**. *BMF2* is expressed throughout plant growth and development.

**Supplementary Figure S2**. Schematics of molecular constructs used in maize transformation.

**Supplementary Figure S3**. Genotyping strategy of the various *Zmbmf2* mutants.

**Supplementary Figure S4**. Silks are produced albeit at a reduced length in *Zmbmf2* mutants.

**Supplementary Figure S5**. *Zmbmf2-5* mutation in A427 inbred reduces silk length.

**Supplementary Figure S6**. Other agronomic traits of *Zmbmf2* mutants.

**Supplementary Table S1**. Segregation analysis of *nptII* marker gene in transgenic *Arabidopsis.*

**Supplementary Table S2**. List of primers.

**Supplementary Table S3**. Segregation analysis of modified *ZmBMF2* transgene in transgenic maize.

## Dive Curated Terms

The following phenotypic, genotypic, and functional terms are of significance to the work described in this paper:

BMF1 Gramene: At2g20635
BMF1 Araport: At2g20635

BMF3 Gramene: At5g05510
BMF3 Araport: At5g05510

MAD2 Gramene: AT3G25980
MAD2 Araport: AT3G25980

BUB3;1 Gramene: At3g19590
BUB3;1 Araport: At3g19590

BUB3;2 Gramene: At1g49910
BUB3;2 Araport: At1g49910

BUB3;3 Gramene: At1g69400
BUB3;3 Araport: At1g69400

BMF2 Gramene: AT2G33560
BMF2 Araport: AT2G33560

MAD1 Gramene: At5g49880
MAD1 Araport: At5g49880

MPS1 Gramene: At1g77720
MPS1 Araport: At1g77720
