# Supplementary material for: The stunt of stunted silk: A novel pollination control mechanism in maize
Source: Plant Physiol. 2026 Jan 28;200(1):kiaf625. doi: 10.1093/plphys/kiaf625 (PMC12851111; doi:10.1093/plphys/kiaf625)
Supplement: kiaf625_Supplementary_Data [file kiaf625_supplementary_data.zip › SupplementaryMethods,Figures, and TableS2.pdf]

Supplementary Data

The Stunt of Stunted Silk: A Novel Pollination Control Mechanism in Maize

Authors: Siddique I. Aboobucker[1*] (صديق أبوبكر), Sidramappa C. Talekar[1, 2], Ursula K. Frei[1],

Bing Yang[3,4], Thomas Lübberstedt[1*]

Affiliations:

[1]Department of Agronomy, Iowa State University; Ames, USA

[2]All India Coordinated Maize Improvement Project, Main Agricultural Research Station, University of Agricultural Sciences; Dharwad, India

[3]Division of Plant Science and Technology, Bond Life Sciences Center, University of Missouri; Columbia, USA

[4]Donald Danforth Plant Science Center; St. Louis, USA

*Corresponding authors. Email: siddique@uky.edu Email: thomasl@iastate.edu

The author responsible for distribution of materials integral to the findings presented in this article in accordance with the policy described in the Instructions for Authors (https://academic.oup.com/plphys/pages/General-Instructions) is Siddique I. Aboobucker (siddique@uky.edu).

Supplementary Methods

Plant materials

Arabidopsis

Arabidopsis thaliana (wild type; Col-0; CS70000) and *bmf2* mutant (Komaki and Schnittger, 2017) seed stocks were obtained from the Arabidopsis Biological Resource Center, Columbus, OH. Seeds were surface sterilized with 70% ethanol followed by 3% sodium hypochlorite and several rinses with sterile water. They were sown in sterile petri dishes containing MS media (Murashige and Skoog, 1962), stratified for 3 days and incubated in a growth chamber with light intensity of 150 µmol m^{-2} s^{-1} for 16h and dark for 8h, at a constant temperature of 23ºC. Germinated seedlings (7-days old) were transferred to pots containing Sunshine LC1 mix and kept at the same growth chamber. Plants with many unopened flower buds were used for transformation by floral dip method (Clough and Bent, 1998). MS media containing kanamycin (50 mg/L) and carbenicillin (500 mg/L) were used for selection of transgenic plants. Oryzalin assays were performed as previously described (Komaki and Schnittger, 2017). Briefly, oryzalin stocks (dissolved in DMSO) were added to MS plates at a concentration of 100 nM or DMSO (control) were used to grow seedlings for 5-days followed by imaging to quantify root length by ImageJ software (Schneider et al., 2012).

Maize

Maize seeds (B104 and A427) were obtained from the North Central Regional Plant Introduction Station (Ames, IA). Transgenic plants were grown in the field in Summer 2020 to 2023 and the GPS co-ordinates are for North Woodruff: 41.990435, -93.690704 or South Woodruff: 41.983747, -93.690569 near Ames, IA. Or in Winter, they were grown in the greenhouse in 2-

gallon pots containing 900MSi potting mix and the conditions were set to 25°C during day and 23°C at night; 16 h light and 8 h dark. Supplemental lights were provided by incandescent bulbs. Standard pollination techniques were used except for the *Zmbmf2* mutants in which silks were manually exposed by cutting open the husks and pollinated.

Vector construction to complement *Arabidopsis bmf2* mutant

To complement *Arabidopsis bmf2* mutant with *ZmBMF2* genomic fragment, it was amplified from B104 genomic DNA using primers *ZmBMF2*-F and *ZmBMF2*-R. *AtBMF2* promoter and terminator fragments were amplified from WT *Arabidopsis* genomic DNA using Pro-*AtBMF2*-F + Pro-*AtBMF2*-R and Term-*AtBMF2*-F + Term-*AtBMF2*-R, respectively (Table S3). These fragments were assembled in *SacI* and *HindIII* digested pCAMBIA2300 vector fragment using NEBuilder HiFi DNA Assembly (New England Biolabs, Ipswich, MA, United States). As a positive control, *AtBMF2* genomic fragment amplified using Pro-*AtBMF2*-F and Term-*AtBMF2*-R primers were ligated to the *SacI/HindIII* pCAMBIA2300 vector using NEBuilder HiFi DNA Assembly. All primers were from Integrated DNA Technologies (Coralville, IA, United States). Plasmid DNA purified from transformed *E. coli* clones resistant to kanamycin were verified by Sanger sequencing at the Iowa State University DNA Facility. Successful clones were transformed to *Agrobacterium* C58C1 strain using freeze-thaw method followed by *Arabidopsis* transformation by the floral dip method (Clough and Bent, 1998).

End-point RT-PCR

Total RNA was extracted from 9-day old *Arabidopsis* seedlings of indicated genotypes grown in MS media with Qiagen RNeasy Plant Mini Kit (Qiagen, Germantown, MD, United States) and treated with rDNaseI (Thermo Fisher, Waltham, MA, United States). First strand cDNA was synthesized using Oligo $(dT)_{20}$ primers and 1 μg of RNA using Superscript III First Strand

cDNA synthesis system (Invitrogen, Carlsbad, CA, United States). Manufacturer's instructions were followed for the above steps. *AtBMF2* and *ZmBMF2* full length transcripts were amplified using respective F and R primers (Table S3) and resolved on 1% Agarose gel electrophoresis. *AtEF1α-A* is the loading control. The expected size of the PCR products are indicated and confirmed by Sanger sequencing.

CRISPR/Cas9 construct for genome editing and complementation in maize

A pair of oligonucleotides (gRNA1-F and gRNA1-R for one guide RNA; gRNA2-F and gRNA2-R for the second guide RNA) complementary each other except 4 nucleotides at their 5′ ends (Table S3) were annealed to obtain double stranded gRNA fragments followed by phosphorylation to add 5′-P groups. gRNA1 fragment was first cloned to the *BtgZ*I digested pENTR-gRNA1 vector followed by a successive cloning of gRNA2 fragment to the *Bsa*I restriction site. Clones were verified by Sanger sequencing and the two gRNA cassettes, each driven by a rice U6 promoter and both together flanked by the *att*L1 and *att*L2 sequences (Figure S2) from the correct clone were Gateway recombined into pGW-Cas9 as previously described (Char et al., 2017). Standard molecular cloning techniques were used.

To complement the *Zmbmf2-3* mutant, the *ZmBMF2* transgene from the A427 inbred line (*ZmBMF2*^{A427}) was chosen since it offers two advantages. One, a SNP at gRNA2 site provides immunity to the CRISPR construct (Figure S2) and the second is a 19 bp indel polymorphism that will discriminate between the B104 and A427 alleles (Figure S3). For assembling a CRISPR-immune modified *ZmBMF2*^{A427} fragment, two fragments were generated by PCR amplification using two primer combinations: *ZmBMF2*^{A427}-F1 + *ZmBMF2*^{A427}-R1 and *ZmBMF2*^{A427}-F2 + *ZmBMF2*^{A427}-R2. These fragments include a ~4 kb promoter region upstream of ATG and ~2 kb terminator region downstream of TAA along with the synonymous

modifications at the gRNA1 site. These two fragments were assembled using NEBuilder HiFi DNA Assembly into *BamHI/EcoRI* pENTR4 vector (Figure S2). The CRISPR construct is expected to express two gRNAs and *Cas9* to mutagenize *ZmBMF2* gene while the modified transgene construct complements the mutation. Both the CRISPR and the complementation constructs were co-transferred into calli from B104 immature embryo by biolistic method at the Iowa State University Plant Transformation Facility. Calli resistant to Glufosinate were transferred to shoot and root regeneration media followed by transferring to soil and obtaining full grown plants. The transformation project yielded six events (#1 to #6) but only two of them survived (#3 and #6) to produce seeds. *Zmbmf2-1, Zmbmf2-2, Zmbmf2-4* and *Zmbmf2-5* were all derived from event #3, while *Zmbmf2-3, Zmbmf2-3/ZmBMF2* and *ZmBMF2* transgene were from event #6. Plants carrying edits were cross-pollinated to segregate away the CRISPR construct with the help of *Cas9* genotyping (Table S3) followed by self-pollination to obtain homozygous mutants and/or *ZmBMF2* transgene.

Phenotypic data collection

Mutant ears were observed to not produce longer silks as the WT throughout the growing season until harvest in all four Summers: 2020 to 2023. Therefore, seeds of the seven genotypes (Figure 1) were planted in 3 rows (20 kernels each) in a randomized design in Summer 2022 and 2023 surrounded by WT plants to provide an abundant pollen source in addition to pollen produced by mutants since they are male fertile. The dates when ears first appear in WT and mutants were recorded. Approx. eight ears per row (39 to 64 ears per genotype; Figure 1) after full length silk emergence (in reference to WT) were harvested from WT and age-matched mutant ears. This approach was taken since silk almost never emerged from the mutants even at the time of harvest. After removing husks, these ears were photographed to measure silk length and ear

length using ImageJ software (Schneider *et al.*, 2012). The remaining ears in the rows were allowed to open-pollinate and harvested at the end of the season to count kernels per ear and kernel row number. At least six *Zmbmf2* mutant ears per genotype per year were pollinated after removing husks to assess fertility. Plant height (from ground up to the start of tassel) and ear height (from the ground to the base of the ear) were measured at physiological maturity in all standing plants in a row.

Statistical analysis

Analysis of variance (ANOVA) was performed to compare the difference in genotypes across two years. For multiple groups comparison, the Tukey test was conducted at a confidence interval of 0.95 with "emmeans" package in R software (version 4.2.0).

Supplementary References

Char, S.N., Neelakandan, A.K., Nahampun, H., Frame, B., Main, M., Spalding, M.H., Becraft, P.W., Meyers, B.C., Walbot, V., Wang, K., et al. (2017). An Agrobacterium-delivered CRISPR/Cas9 system for high-frequency targeted mutagenesis in maize. Plant Biotechnology Journal **15**:257-268. 10.1111/pbi.12611.

Clough, S.J., and Bent, A.F. (1998). Floral dip: a simplified method for *Agrobacterium*-mediated transformation of *Arabidopsis thaliana*. The Plant Journal **16**:735-743. 10.1046/j.1365-313x.1998.00343.x.

Komaki, S., and Schnittger, A. (2017). The spindle assembly checkpoint in Arabidopsis is rapidly shut off during severe stress. Developmental Cell **43**:172-185. 10.1016/j.devcel.2017.09.017.

Murashige, T., and Skoog, F. (1962). A revised medium for rapid growth and bio assays with tobacco tissue cultures. Physiologia Plantarum **15**:473-497. 10.1111/j.1399-3054.1962.tb08052.x.

Schneider, C.A., Rasband, W.S., and Eliceiri, K.W. (2012). NIH Image to ImageJ: 25 years of image analysis. Nature Methods **9**:671-675. 10.1038/nmeth.2089.

Supplementary Figures

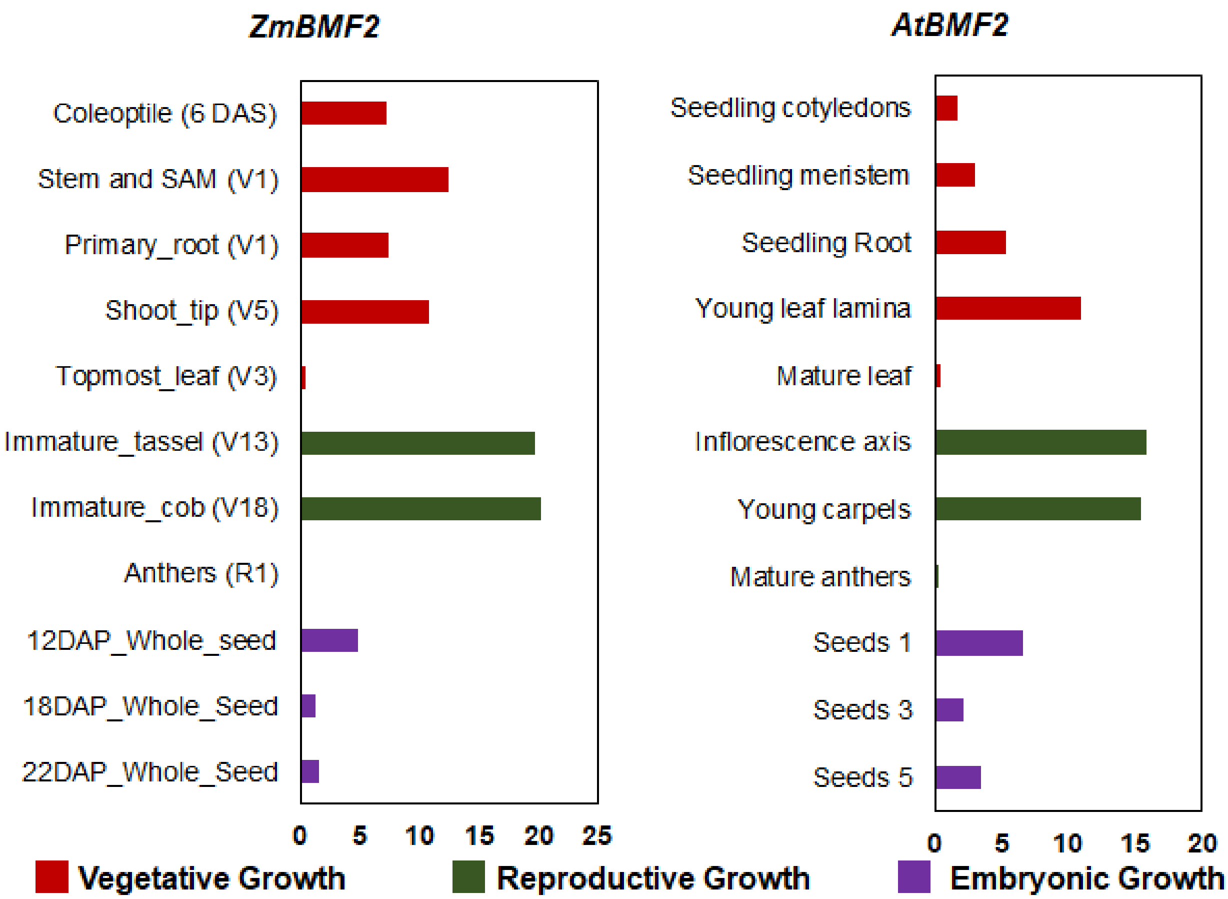

Figure S1. *BMF2* is expressed throughout plant growth and development. Publicly available gene expression data for *ZmBMF2* and *AtBMF2* from select tissue type as indicated are shown. Tissue types were manually grouped into three different growth phases as indicated. DAS, days after sowing; SAM, shoot apical meristem; DAP, days after pollination.

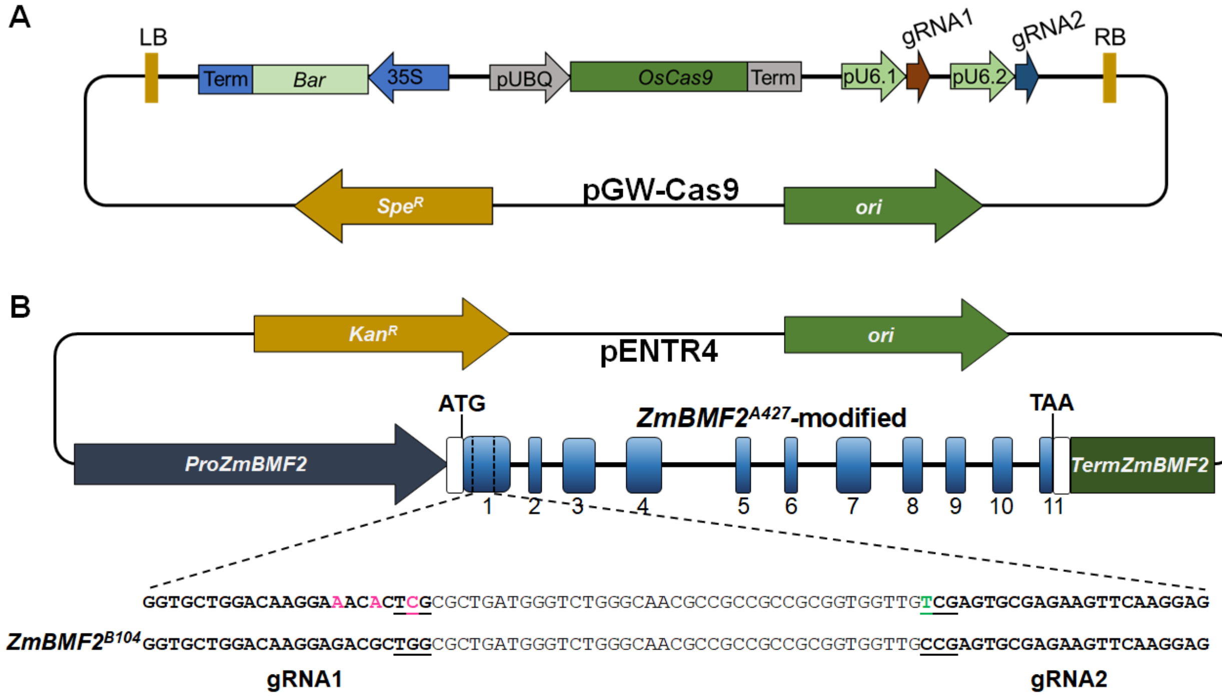

Figure S2. Schematics of molecular constructs used in maize transformation. A) pGW-Cas9 plasmid (Char et al., 2017) carrying the CRISPR machinery to edit *ZmBMF2* gene using gRNA1 and gRNA2, which are driven by pU6.1 and pU6.2 promoters, respectively. The *OsCas9* gene is flanked by the *ZmUbi* promoter and an OCS terminator, while *Bar* resistance gene is placed between 35S promoter and NOS terminator. LB, left border; RB, right border; Spe^R, spectinomycin resistance; *ori*, origin of replication. **B)** pENTR4 vector carrying the modified genomic fragment of *ZmBMF2* from A427 inbred line to complement the *Zmbmf2* mutants is shown. Exons are shown as boxes and their numbers shown below, whereas introns are denoted by lines. A ~4-kb fragment upstream of ATG is considered the native promoter and a ~2-kb fragment downstream of TAA is the native terminator of *ZmBMF2*. In the close-up sequence view, gRNAs are indicated by bold face and PAM underlined. The "CRISPR-immune" synonymous modifications made in gRNA1 site in $ZmBMF2^{A427}$ is indicated by red color, while the natural variation at the PAM site of gRNA2 in A427 inbred is shown in green color. Kan^R, kanamycin resistance; *ori*, origin of replication.

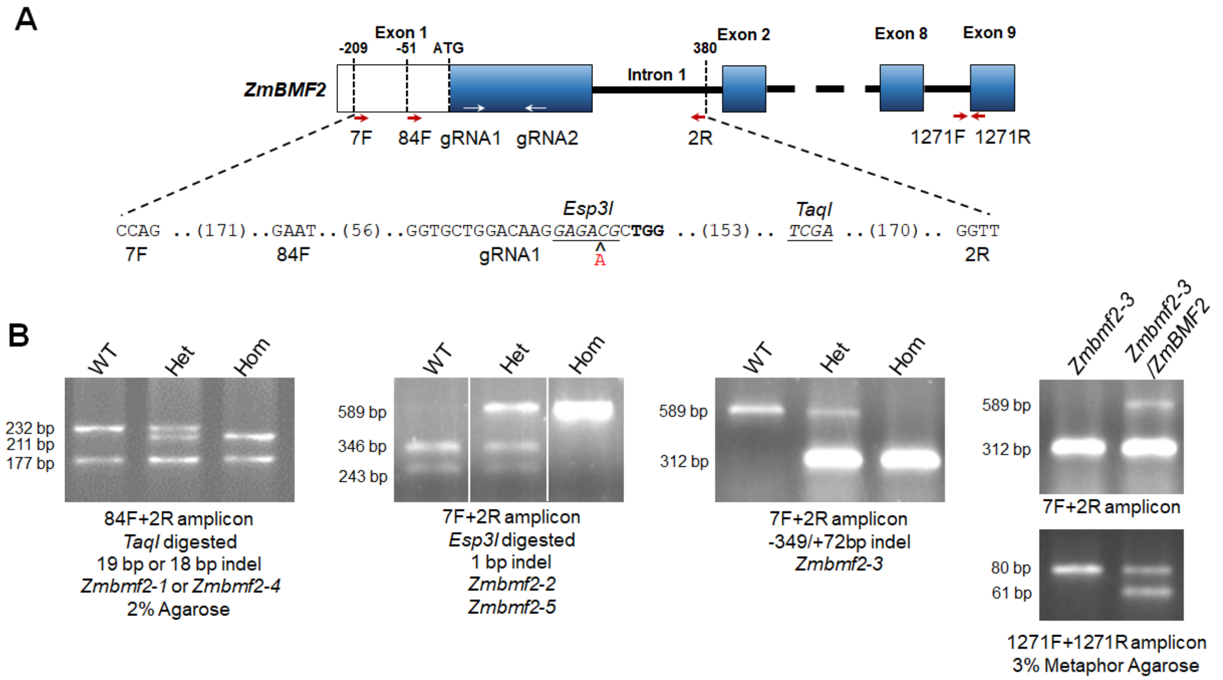

Figure S3. Genotyping strategy of the various *Zmbmf2* mutants. A) Cartoon representation of the *ZmBMF2* gene from B104 showing a selected number of exons and introns in boxes and lines, respectively. The unfilled box before ATG of Exon 1 denotes the 5' UTR. The numbers above exons and introns denote the nucleotide distance in reference to the ATG. Primer sites used in PCR genotyping are indicated by short red arrows, while the gRNA sites are in white arrows. A 19 bp indel polymorphism between B104 and A427 alleles are shown by the primer sites between Exons 8 and 9. In the sequence view, partial sequences of the primer sites are marked with their names, full 20 bp sequence of the gRNA1 site is shown along with the PAM site in bold. *Esp3I* and *TaqI* restriction enzyme recognition sites are in italics, the 1 bp insertion position in *Zmbmf2-2* mutant is denoted by a Cap symbol and the inserted nucleotide in red color. Numbers in parentheses indicate the number of nucleotides not shown in the sequence. **B)** Agarose gel electrophoresis pictures exemplifying the genotyping of the various *Zmbmf2* mutants as indicated. The presence of *Zmbmf2-3* mutation and *ZmBMF2* transgene were identified by using two sets of PCRs as shown. All gels are 1% Agarose unless noted otherwise. WT, wild type and no mutation; Het, mutation heterozygous; Hom, mutation homozygous.

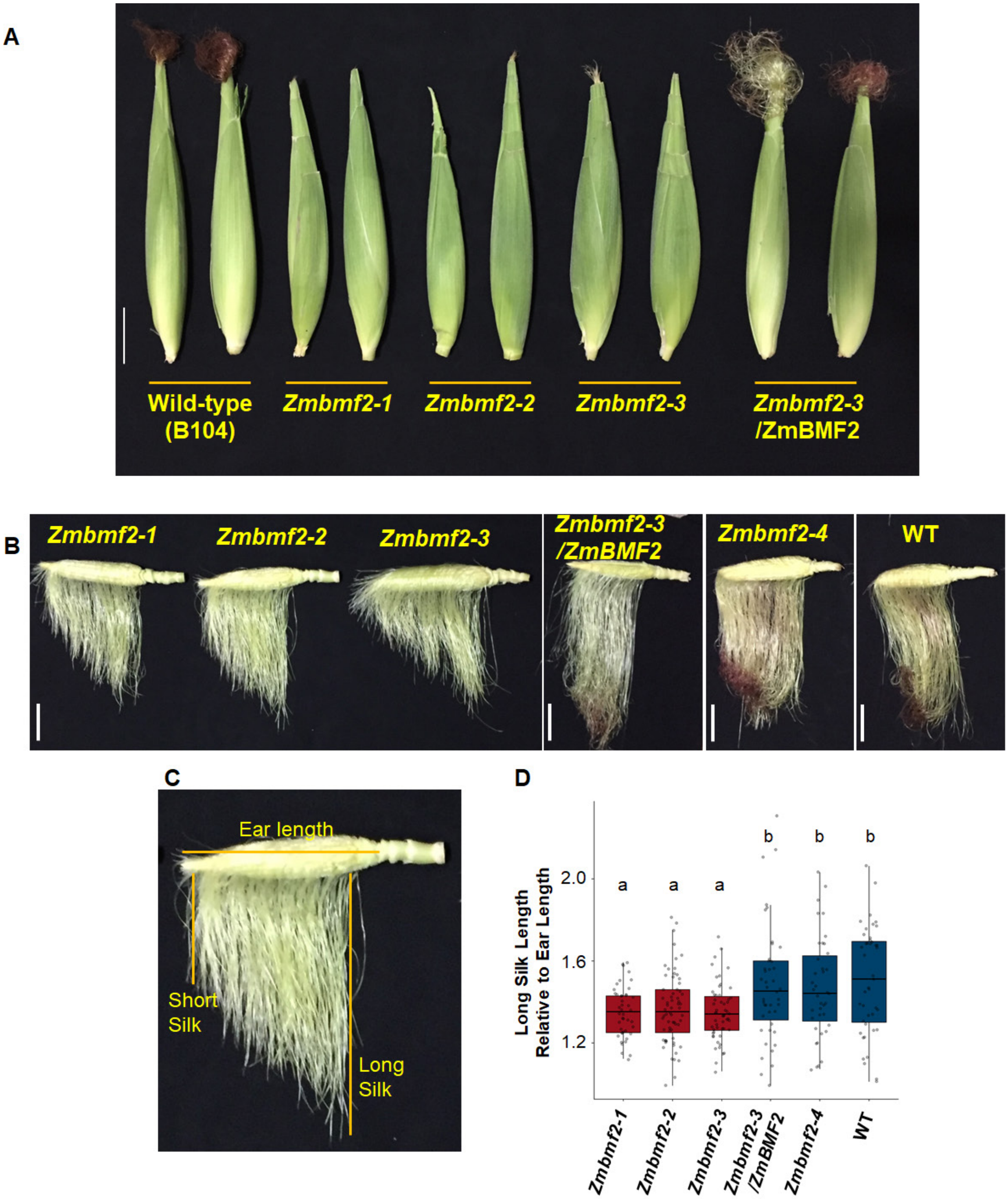

201

202

203 **Figure S4. Silks are produced albeit at a reduced length in *Zmbmf2* mutants. A)** Additional
204 representative photographs of ears from the various *Zmbmf2* mutants obtained during the project

in summer 2020 to 2022. **B)** A view of the ears from the various genotypes with their husks removed show the presence of silks but at a reduced length in the *Zmbmf2-1*, *Zmbmf2-2 and Zmbmf2-3* mutants compared to *Zmbmf2-4, Zmbmf2-3/ZmBMF2* and WT. Scale bar, 5 cm. **C)** Figure exemplifying the measurements of ear length, “short silk” and “long silk” lengths at the tip and base ends, respectively, are shown, which are used to calculate relative silk lengths (as in D and Fig. 2E). **D)** Quantification of long silk length relative to ear length from the mutants, complemented line and WT. Box plots show the distribution of data points with the median as a center line. The upper (75th percentile) and lower (25th percentile) quartiles are shown by the bounds of boxes, and the whiskers represent the highest and lowest observations. Different letters show statistical significance by Tukey test at a 95% confidence interval.

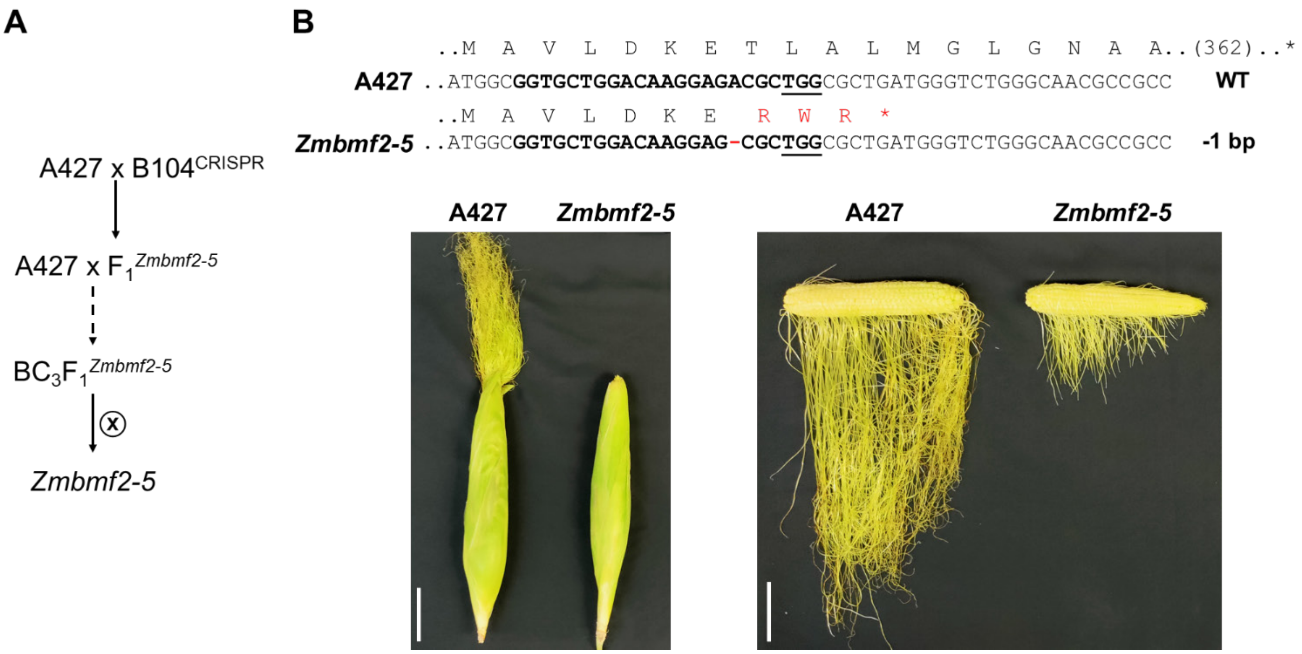

Figure S5. *Zmbmf2-5* mutation in A427 inbred reduces silk length. A) Breeding strategy used to obtain *Zmbmf2-5* mutant in the A427 background is shown. Transformed B104 carrying Cas9 and gRNAs was used to introduce the CRISPR reagents into A427. Dashed arrows indicate multiple generations. **B)** Partial nucleotide sequence of the *ZmBMF2* gene in A427 and *Zmbmf2-5* are shown; gRNA1 site is in bold face and PAM site is underlined. The 1 bp deletion is indicated by a dash and the impact at the amino acid level compared to A427 are also shown in red font. Representative photographs of ears during silking stage from the A427 and *Zmbmf2-5* are presented. Scale bar, 5 cm.

242

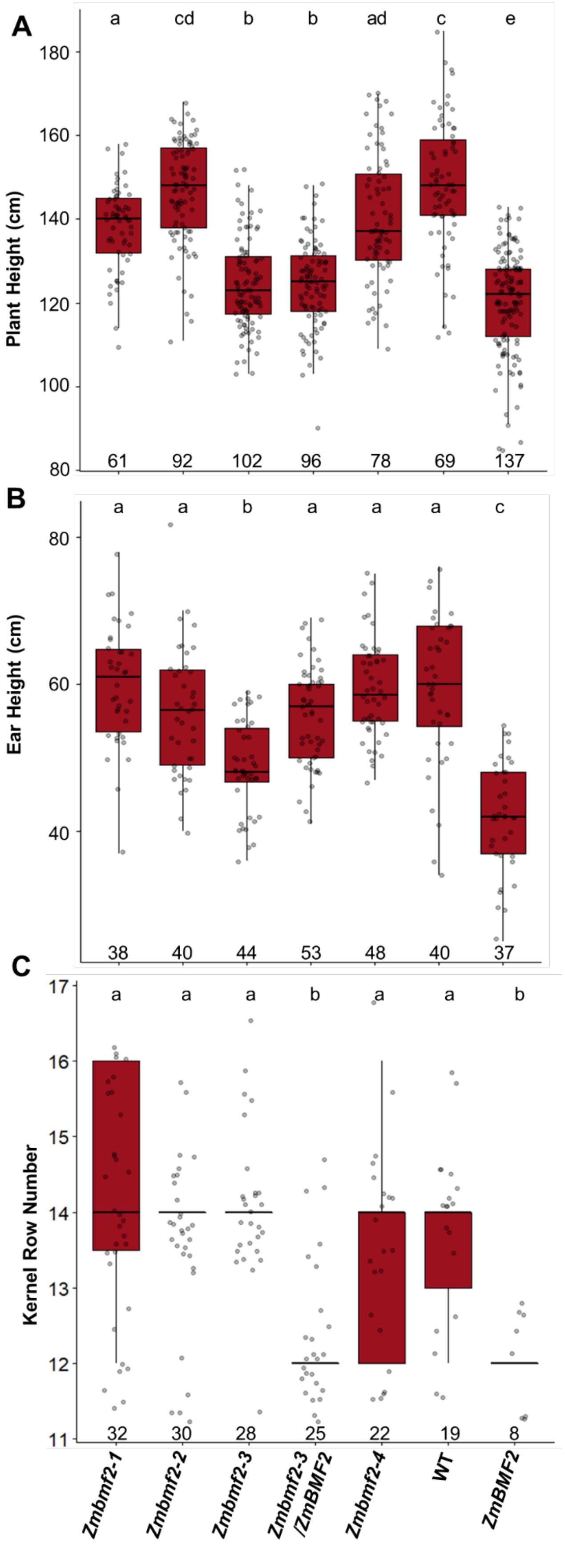
A
Plant Height (cm)
a cd b b ad c e
61 92 102 96 78 69 137
B
Ear Height (cm)
a a b a a a c
38 40 44 53 48 40 37
C
Kernel Row Number
a a a b a a b
32 30 28 25 22 19 8
Zmbmf2-1
Zmbmf2-2
Zmbmf2-3
Zmbmf2-3 /ZmBMF2
Zmbmf2-4
WT
ZmBMF2

Figure S6. Other agronomic traits of *Zmbmf2* mutants. A-C) Measurements of the three different agronomic traits: plant height **(A)**, Ear height **(B)** and kernel row number **(C)** are shown. Data in A are from Summer 2022 and 2023 and the data in B and C are from Summer 2023 only. Box plots show the distribution of data points with the median as a center line and each dot represents plant height (A), ear height (B) or kernel row number (C). The upper (75th percentile) and lower (25th percentile) quartiles are shown by the bounds of boxes and the whiskers represent the highest and lowest observations. Different letters show statistical significance by Tukey test at a 95% confidence interval. The number of individuals per genotype is indicated at the bottom of each panel. *ZmBMF2* indicates the line in B104 background carrying only the modified *ZmBMF2* transgene from A427 (Figure S2).

Table S2. List of primers.

Primer name	Primer sequence (5’ to 3’)	Purpose
Pro-*AtBMF2*-F	CCATGATTACGAATTC*GAGCTC*GTTTACCTCCGGTGTTATCGG	Cloning *AtBMF2* or *ZmBMF2* genomic sequences to complement oryzalin phenotype[1]
Pro-*AtBMF2*-R	CATCGTCGTTTCTTCGAGCAAAT	
ZmBMF2-F	ATTTGCTCGAAGAAACGACGATGGCGGCGGCGGAAGAGAT	
ZmBMF2-R	TGTAGAGAAGAAAGTCCAATATCAGATTACCTAAGCCTGCTCAGG	
Term-*AtBMF2*-F	TGATATTGGACTTTCTTCTCTACA	
Term-*AtBMF2*-R	AACGACGGCCAGTGCC*AAGCTT*GAACCAATGCCAATGTTTTGTTC	
AtBMF2-F	ATGGCAGCCGAAACGAAGGT	RT-PCR
AtBMF2-R	TCATCGTAGGAAGCTGTTGG	
AtEF1α-A_F	TGAGCACGCTCTTCTTGCTTTCA	
AtEF1α-A_R	GGTGGTGGCATCCATCTTGTTACA	
gRNA1-F	tgttGGTGCTGGACAAGGAGACGC	gRNA construction[2]
gRNA1-R	aaacGCGTCTCCTTGTCCAGCACC	
gRNA2-F	gtgtgCTCCTTGAACTTCTCGCACT	
gRNA2-R	aaacAGTGCGAGAAGTTCAAGGAGc	
ZmBMF2^{A427}-F1	GAACCAATTCAGTCGACT*GGATCC*ACTAGCGGTCCTCAGTT	Cloning modified *ZmBMF2*^{A427} transgene to complement silk length[3]
ZmBMF2^{A427}-R1	CGCGAGTGTTTCCTTGTCCAGCACCGCCA	
ZmBMF2^{A427}-F2	CTGGACAAGGAAACACTCGCGCTGATGGGTCTGGGCAAC	
ZmBMF2^{A427}-R2	GATATCTCGAGTGCGGCCGC*GAATTC*AGTTGTGCGCATACATCAGG	
OsCas9F	GGGTAATGAACTCGCTCTGC	Genotyping CRISPR mutants
OsCas9R	TGGCGTCAAGAACTTCCTTTG	
84F	AACGGTCGAATCTCCTCGAAT	

7F	CCAGCCAAGCAAAACATAGC	
2R	TGTGTGTTCATCGTTCGGTT	
1271F	TGCACCGAGGACAACTATTCAG	
1271R	GCTCCTAACTTCTGTGGAATCTGT	

[1] Overlapping sequence for NEBuilder HiFi DNA Assembly are underlined. Sequences in Pro-F and Term-R overlap to *SacI* and *HindIII* (shown in italics) digested sites in pCAMBIA2300 vector, respectively; the ones in Pro-R and *ZmBMF2*-F; and *ZmBMF2*-R and Term-F overlap to each other.

[2] *BtgZI* and *BsaI* overhang sites aiding in the ligation of gRNA1 and gRNA2, respectively, to pENTR-gRNA1 vector is in lowercase.

[3] Overlapping sequences for NEBuilder HiFi DNA Assembly are underlined. Sequences in F1 and R2 overlap to *BamHI* and *EcoRI* (shown in italics) digested sites in pENTR4 vector, respectively; the ones in R1 and F2 overlap to each other. The nucleotides modified to introduce synonymous mutations are marked in red.
