# Supplementary material for: The stunt of stunted silk: A novel pollination control mechanism in maize
Source: Plant Physiol. 2026 Jan 28;200(1):kiaf625. doi: 10.1093/plphys/kiaf625 (PMC12851111; doi:10.1093/plphys/kiaf625)
Supplement: kiaf625_Supplementary_Data [file kiaf625_supplementary_data.zip › TableS1.pdf]

Table S1. Segregation analysis of *nptII* gene in T_2 and T_3 families transformed with *ZmBMF2* or *AtBMF2* in *bmf2* background

Molecular construct	Generation	Line-plant number	Seeds grown in kanamycin (50 mg/L)			$\chi^{2\ a}$	*P* value
			Total	Resistant (R)	Susceptible (S)		
ProAtBMF2::ZmBMF2	T2	**Parental Line 2-4** [b]	75	57	18	0.04	0.84
	T3	2-4_P2	59	59	0	19.67	0.00
	T3	2-4_P3*	59	59	0	19.67	0.00
	T3	2-4_P4	54	36	18	2.00	0.16
	T3	2-4_P6	57	43	14	0.01	0.94
	T3	2-4_P7	59	45	14	0.05	0.82
	T3	2-4_P11	55	43	12	0.30	0.59
	T3	2-4_P12	51	31	20	5.50	0.02
	T3	2-4_P15	57	45	12	0.47	0.49
ProAtBMF2::ZmBMF2	T2	**Parental Line 2-6** [b]	68	52	16	0.08	0.78
	T3	2-6_P2	56	39	17	0.86	0.35
	T3	2-6_P3	58	44	14	0.02	0.88
	T3	2-6_P5	58	47	11	1.13	0.29
	T3	2-6_P6	58	40	18	1.13	0.29
	T3	2-6_P8	58	45	13	0.21	0.65
	T3	2-6_P9	57	43	14	0.01	0.94
	T3	2-6_P11*	59	59	0	19.67	0.00
	T3	2-6_P12	60	42	18	0.80	0.37
ProAtBMF2::AtBMF2	T2	**Parental Line 1-3** [b]	69	53	16	0.12	0.73
	T3	1-3_P1	37	26	11	0.44	0.51
	T3	1-3_P2	45	45	0	15.00	0.00
	T3	1-3_P3*	40	40	0	13.33	0.00
	T3	1-3_P5	42	41	1	11.46	0.00
	T3	1-3_P6	40	29	11	0.13	0.72
	T3	1-3_P10	43	34	9	0.38	0.54
	T3	1-3_P13	56	46	10	1.52	0.22
	T3	1-3_P14	65	55	10	3.21	0.07
ProAtBMF2::AtBMF2	T2	**Parental Line 2-1** [b]	60	45	15	0.00	1.00
	T3	2-1_P1	47	37	10	0.35	0.56
	T3	2-1_P4	54	44	10	1.21	0.27
	T3	2-1_P5	55	37	18	1.75	0.19
	T3	2-1_P6*	50	49	1	14.11	0.00
	T3	2-1_P11	52	44	8	2.56	0.11
	T3	2-1_P12	55	40	15	0.15	0.70
	T3	2-1_P14	45	36	9	0.60	0.44
	T3	2-1_P16	54	38	16	0.62	0.43

[a] Ratio tested was 3:1 with critical $\chi 2$ value being 3.841

[b] Segregation analysis of Parental T_2 families, the progenitor of the analyzed T_3 families

* and yellow highlighting indicate single insertion homozygous plants that were used in oryzalin assays
