# Supplementary material for: The stunt of stunted silk: A novel pollination control mechanism in maize
Source: Plant Physiol. 2026 Jan 28;200(1):kiaf625. doi: 10.1093/plphys/kiaf625 (PMC12851111; doi:10.1093/plphys/kiaf625)
Supplement: kiaf625_Supplementary_Data [file kiaf625_supplementary_data.zip › TableS3.pdf]

Table S3.Segregation analysis of *ProZmBMF2::ZmBMF2*^{modified} transgene in transformed T2, T3 and T4 families using PCR and agarose gel electrophoresis

Molecular construct	Generation	Line-plant number	Number of seedlings tested			$\chi^{2\ b}$	*p* value
			Total	Trangene +	Transgene -		
ProZmBMF2::ZmBMF2^{modified}	T2	**6-15** [b]	20	15	5	0.00	1.00
	T3	6-15_P4	27	17	10	2.09	0.15
	T3	6-15_P7	14	11	3	0.10	0.76
	T4	6-15_P7_P11*	15	15	0	5.00	0.03
	T4	6-15_P7_P12	15	10	5	0.56	0.46
	T4	6-15_P7_P13	14	10	4	0.10	0.76
	T4	6-15_P7_P22	16	8	8	5.33	0.02
	T4	6-15_P7_P23	14	14	0	4.67	0.03
	T4	6-15_P7_P25	15	10	5	0.56	0.46

[a] Ratio tested was 3:1 with critical $\chi 2$ value being 3.841

[b] Segregation analysis of Parental T2 families, the progenitor of the analyzed T3 families

* and yellow highlighting indicate single insertion homozygous plants used in silk length analysis for the complementation experiment
